# Optimization of the Ultrasound-Assisted Extraction of Phenolic Compounds from *Brosimum alicastrum* Leaves and the Evaluation of Their Radical-Scavenging Activity

**DOI:** 10.3390/molecules22081286

**Published:** 2017-08-07

**Authors:** Mariel Gullian Klanian, Montserrat Terrats Preciat

**Affiliations:** Experimental Unit, University Marist of Mérida, Periférico Norte Tablaje Catastral 13941, Carretera Mérida-Progreso, 97300 Mérida, Yucatán, Mexico; mterrats@marista.edu.mx

**Keywords:** *Brosimum alicastrum* leaf, ultrasound assisted extraction, response surface methodology, total phenolic content, monomeric anthocyanin, radical-scavenging activity

## Abstract

In order to maximize the yield of the total phenolic content (TPC) and total monomeric anthocyanin (TMA) from *Brosimum alicastrum* leaf and to study the radical-scavenging activity, a three-level three-factor Box–Behnken design (BBD) was used to determine the optimal points for ultrasound-assisted extraction (UAE). In this study, we analyzed the extraction time (10, 20, and 30 min), temperature (28, 30, and 32 °C), and probe sonication power (40%, 28 W/cm^2^; 60%, 51 W/cm^2^; and 80%, 74 W/cm^2^). Analysis of variance (ANOVA) indicated that the sonication power plays a significant role in the extraction of phenolic compounds. An increase in time and temperature resulted in a decrease in the yield, in particular, of the TMA group. DPPH was found to be a better indicator of radical-scavenging activity than ABTS. The predicted TPC and TMA optimum levels (45.18 mg GAE/g and 15.16 mg CyE/100 g) were obtained at 28 °C, 80%, and 20–10 min. DPPH obtained a maximum value (67.27 μmol TE/g) under same optimization conditions. The RSM confirmed that TPC and TMA enhanced the antioxidant activity when subjected to low temperature (28 °C), extraction time less than 20 min, and higher sonication power (74 W/cm^2^), and hence achieving the better DPPH scavenging activity.

## 1. Introduction

*Brosimum alicastrum* Swartz, which used to be cultivated and used as a subsistence food by the ancient Mayan Civilization from 300 to 900 A.C., is a tree member of the Moraceae family of edible botanicals [1]. The primary growing region of *B. alicastrum* ranges from the southern Mexico to Bolivia, but it can also be found in the Caribbean, Cuba, Trinidad, and Jamaica [2]. The fruit contains an edible seed, generally called Maya nut, which may be used as dried or roasted, ground into powder, and incorporated into baked goods. The leaves are mostly used as a potential complement of grazing in the production animals, though for a few years several products are being developed and commercialized for human feeding with an emphasis on their medicinal properties [3]. To the best of our knowledge, the presence of bioactive compounds (BCs) in *B. alicastrum* leaves has never been explored.

BCs are defined as non-nutritional substances that are found in very low concentrations in foods and intervene in the secondary metabolism of vegetables. BCs from seeds, roots, and leaves of the plant have recently gained high a popularity because of their significant positive effects on human health, reducing the risk of inflammatory diseases [4,5,6]. The most common BCs in plant extracts are natural antioxidants, particularly polyphenols such as phenolic acids, flavonoids, anthocyanins, and tannins [7,8].

The optimization of solvent extraction methods in plants has been studied in order to obtain extra yield and better-quality index of the product under the optimum extraction conditions [9]. Hence, ultrasound-assisted extraction (UAE) is a valuable procedure that has been used by several authors to obtain an exhaustive phytochemical recovery of raw and firm plant materials within a short period of time. The main advantage of UAE is the reduction in the extraction time with a low solvent consumption and low temperature, which helps to avoid thermal damages in the extraction of heat-labile products and therefore preserve the structural and molecular properties [10,11]. Generally, the enhanced extraction of total phenols during sonication is caused by the breakage of the vegetal matrix cells by ultrasound waves and thus releasing the cell contents into the extraction medium [12].

The extraction time and the ultrasound power were optimized by previous authors, as important parameters affecting the extraction yield of phenolic compounds in plant leaves [13,14]. Generally, increasing time and temperature promote analytic solubility. However, the phenolic compounds are generally degraded or undergo undesirable reactions such as enzymatic oxidation by extended extraction times and high temperatures [15]. Despite knowing the effect of temperature on phenolic compounds, this parameter has been less explored in particular for UAS-extracted leaves. Using traditional methanol extraction, the effect of temperature on the yield of TPC and free radical scavenging activity was investigated for the leaf extract of *Gynura procumbens* [16]. From UAS extracts, the effect of temperature was studied and optimized in fruit (olive pomace and peach) under the concept that the extraction temperatures interacting with other parameters affect the recovery of phenolic compounds [17,18].

Extract recovery is influenced not only by sonication time and temperature, but also by ultrasonic potency and wave distribution. In particular, the sonication power and amplitude of the probe acting simultaneously on the extract are considered to be very important factors for sonication results [19]. The parameters such as amplitude setting, viscosity, temperature, and sample volume should remain consistent to reproduce the results. The probe sonicator is designed to deliver constant amplitude. Additional power is delivered by the power supply to ensure that the excursion at the probe tip remains constant as the resistance to the movement of the probe increases. Using a more powerful power supply does not deliver more power into the liquid. On the contrary, it is the resistance to the movement of the probe that determines how much power must be delivered into the sample. A sensing network continuously monitors the output requirements, and automatically adjusts the power to maintain the amplitude at the preselected level [20].

In order to achieve higher extraction yields, a model is required for the optimization of the most relevant parameters, mainly for plants where there is not enough research available. The mathematical technique called response surface methodology (RSM) is an effective tool to find the optimal conditions for the process when many parameters and their interactions may affect the desired response. The RSM technique is applied to optimize the extraction conditions of the phenolic content and antioxidant activity obtained from fruits, leaves, and seeds of several vegetables [18,21,22]. Thus, the aim of this study was to optimize the process variables of the UAE for *B. alicastrum* leaves in order to obtain the maximum total phenolic content (TPC) and monomeric anthocyanins (TMA) with high radical-scavenging activities (DPPH and ABTS). The Box–Behnken design (BDD) of RSU was employed to optimize and study the interaction effects of the process variables, such as sonication power (*X*_1_), extraction time (*X*_2_), and temperature (*X*_3_).

## 2. Results and Discussion

### 2.1. Response Surface Methodology for Optimization of UAE

In order to identify the optimal level and the effect of process variables on the responses, the Box–Behnken design (BBD) with 1 replicate was employed. Three levels (high, middle, and low), i.e., sonication power (28 W/cm^2^, 51 W/cm^2^, and 74 W/cm^2^), extraction time (10, 20, and 30 min), and temperature (28, 30, and 32 °C) were used to study the chosen variables. Fifteen experiments were carried out in duplicate by using the BBD design. The responses of TPC, TMA, ABTS, and DPPH scavenging activities are presented in Table 1.

The data obtained from the experiments were subjected to multiple regression analysis for estimating the coefficients to represent the second-order polynomial model. Table 2 presents the results of ANOVA for each independent variable and their interactions.

### 2.2. Response Surface Analysis of TPC

**Figure 1:**
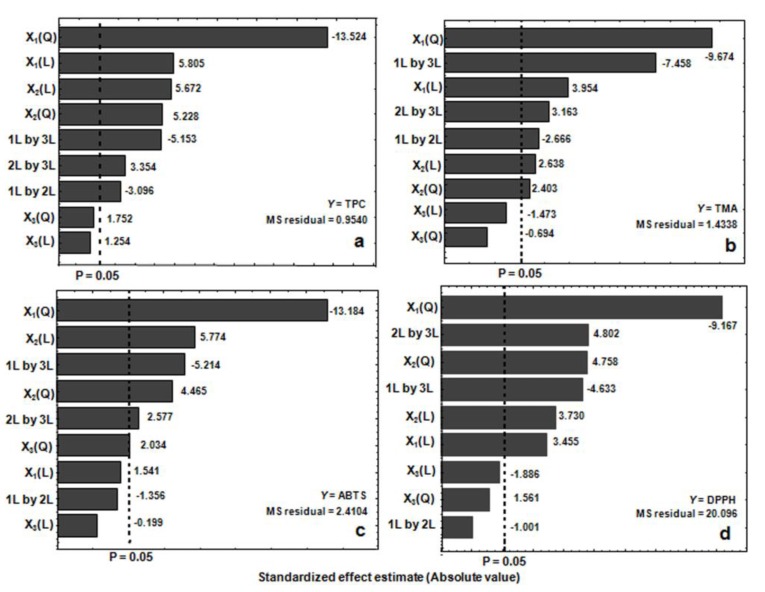
Pareto chart of the standardized effects of the three factors and three levels of Box–Behnken design for *Brosimum alicastrum* leaf-extract. (**a**) total phenolic content; (**b**) total monomeric anthocyanin; (**c**) ABTS; (**d**) DPPH. The vertical dashed line indicates the significance level at *p* = 0.05. The *t*-test value for each effect is shown in the Pareto chart by horizontal columns.

**Figure 2:**
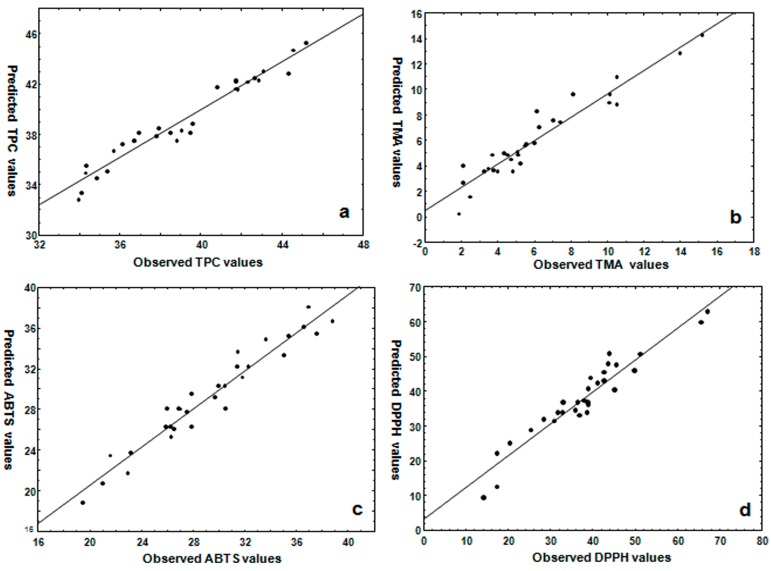
Scatterplot of predicted and observed value of (**a**) total soluble phenol content (TPC); (**b**) total monomeric anthocyanin (TMA); (**c**) ABTS; and (**d**) DPPH of the leaf extract of *Brosimum alicastrum*.

ANOVA statistics for TPC explained that the regression model was significant (*p* < 0.001). Considering *p*-value < 0.05, the linear and the quadratic effect of amplitude *(X*_1_) and time (*X*_2_), and the interactive effect between the three independent variables (*X*_1_, *X*_2_, *X*_3_) was significant for TPC yield. The quadratic effect of the amplitude (*X*_1_^2^) and the linear effect of the time (*X*_2_) were significant at *p* < 0.001. The above-mentioned factors were extended beyond the reference line of the Pareto chart at a significance level of *α* = 0.05 (Figure 1a). The LOF *p*-value of 0.7945 implies that the model fits well and therefore it is adequate to explain the data. The adjusted regression coefficient (R^2^ = 0.9478) was in reasonable agreement with the experimental results, indicating 94.8% of the variability can be measured by the model (Table 2). Figure 2a shows the scatterplot of the predicted TPC values versus the observed values. The predictive equation obtained after neglecting the nonsignificant terms for TPC is expressed as follows:

*Y*_TPC_ = −130.9752 + 0.05444(*X*_1_) − 0.5267(*X*_2_) + 0.0122(*X*_1_^2^) − 0.0188(*X*_2_^2^) − 0.1574(*X*_3_^2^) − 0.0053(*X*_1_*X*_2_) − 0.0445(*X*_1_*X*_3_) + 0.0579(*X*_2_*X*_3_) (1)

**Figure 3:**
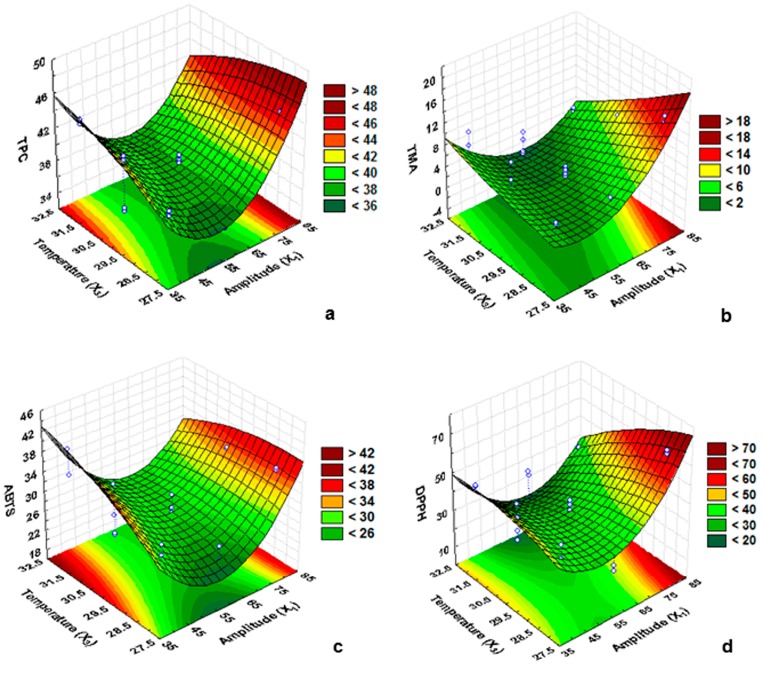
Response surface plot of Box–Behnken design showing the mutual effect of amplitude, time, and temperature on the yield of (**a**) total soluble phenol content (TPC); (**b**) total monomeric anthocyanin (TMA); (**c**) ABTS; and (**d**) DPPH of the leaf extract of *Brosimum alicastrum*.

**Figure 4:**
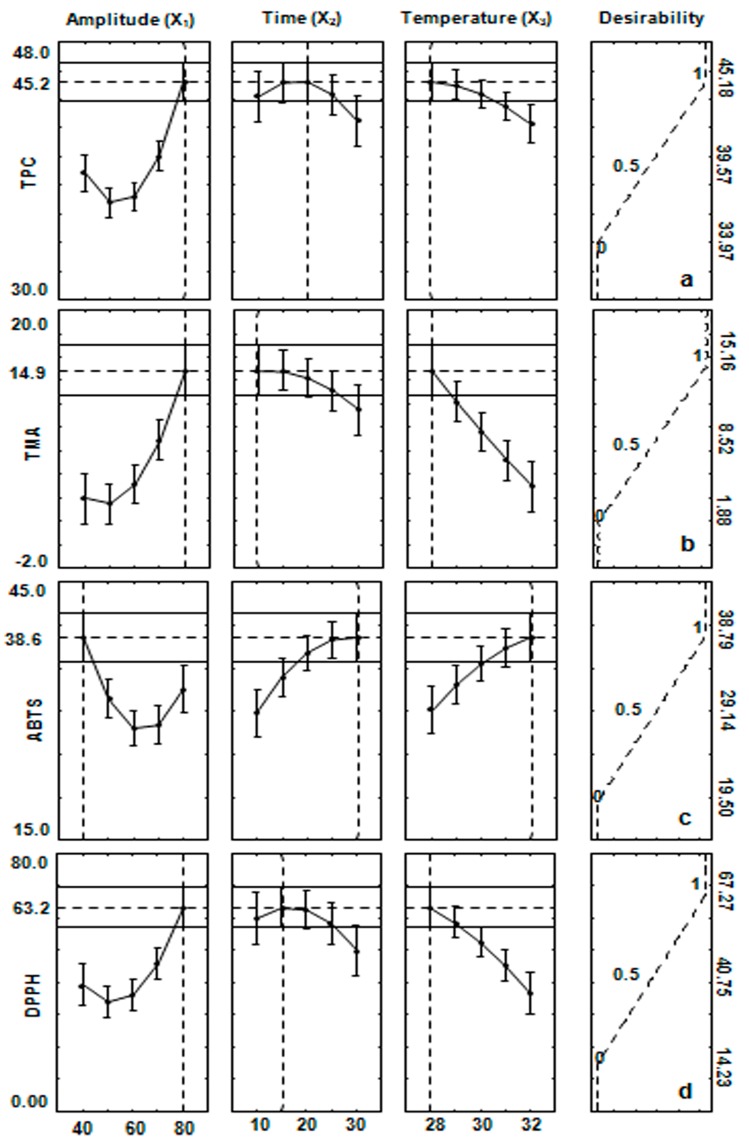
Profiles for predicted values and desirability function for the average recovery of (**a**) total soluble phenol content (TPC); (**b**) total monomeric anthocyanin content (TMA); (**c**) ABTS, and (**d**) DPPH of the leaf extract of *Brosimum alicastrum*. Dashed lines indicate the optimization values.

The overall effect of the independent variables (*X*_1_*X*_3_) on TPC can be seen in the 3D response surface plot as a representation of the polynomial equation obtained from the experimental data (Equation (1); Figure 3a). An increase in TPC was recorded at high amplitude and low temperature. The optimal point determined by the desirability surface contour plot indicated that high yield is obtained when the extract is subjected to the extraction conditions of 80% amplitude (74 W/cm^2^), 20 min, and 28 °C. The desirability of 1.0 was assigned for a maximum TPC of 45.18 mg GAE/g, 0.0 was assigned for a minimum TPC of 39.57 mg GAE/g, and 0.5 was assigned for the mid TPC value of 33.97 mg GAE/g (Figure 4a).

The extraction conditions definitely influenced metabolite recovery at a particular time and temperature. Davidov-Pardo et al. [15] reported that thermal treatments affect the stability of the extract in a different manner depending on the phenolic profile of each extract. When the leaves of *Cratoxylum formosum* ssp. formosum were submitted under optimal UAE conditions of 45 °C for 20–50 min, they produced a maximum TPC yield (41.75 mg GAE/g) [23]. The maximum TPC yield obtained for *C. formosum* was similar to that obtained for *B. alicastrum*, but it was obtained by subjecting the sample to 17 °C higher temperature than that employed in this study. Nevertheless, the methanol extract of *Urtica dioica* L. leaves only reached a maximum TPC of 8.90 mg GAE/g under optimal UAE conditions of 60 W of sonication power, 25 °C, and 38 min [24]. In conclusion, we agree that the temperature can affect differently depending on the type of the sample and possibly the phenolic profile of the extract.

### 2.3. Response Surface Analysis of TMA

Table 2 presents the ANOVA statistics for TMA. The RSU model was significant at a *p*-value of 0.02. The results indicated that the amplitude (*X*_1_, *X*_1_^2^; *p* < 0.01) and the interactive effects between the amplitude and temperature (*X*_1_*X*_3_; *p* < 0.01) and the time and temperature (*X*_2_*X*_3_; *p* < 0.01) were the main factors that influenced the TMA yield (Figure 1b). The LOF *p*-value of 0.1518 was not significant, and therefore the model fits well to the data. Figure 2b shows the scatterplot of the predicted TMA values versus the observed values. The adjusted R^2^ indicated that 87.37% of the variability was shown by the model (Table 2). The predictive equation obtained after neglecting the nonsignificant variables for TMA is expressed as follows:

*Y*_TMA_ = −0.02000 + 1.2615(*X*_1_) − 1.1672(*X*_2_) + 0.0107(*X*_1_^2^) − 0.0106(*X*_2_^2^) − 0.0056(*X*_1_*X*_2_) − 0.0789(*X*_1_*X*_3_) + 0.0669(*X*_2_*X*_3_) (2)

The effect of *X*_1_ (amplitude) and *X*_3_ (temperature) on TMA is shown in 3D response surface plots as a representation of the polynomial equation (Equation (2)). An increase in TMA was recorded with an increase in the amplitude at low temperature (Figure 3b). Based on the desirability score of 1.0, the yield of TMA was optimized at 15.16 mg CyE/100 g under the optimized conditions of 80% amplitude (74 W/cm^2^), 10 min, and 28 °C (Figure 4b).

TMA belongs to the flavonoid family, which is the most common phenolic group widely distributed in plant tissues. As a part of the phenolic compounds, monomeric anthocyanin was optimized under the same conditions of the group, and the concentrations of both compounds were highly correlated (R = 89.1; Table 3). The conditions for achieving the maximum yield of TMA were similar under the conditions that maximized the extraction of TPC, particularly the sonication power and temperature. The extraction time was the only parameter that slightly differed, e.g., a 10-min increase in the extraction time caused a decrease in the TMA yield (Figure 4b).

The fact that the magnitude and duration of heating have a strong influence on anthocyanin stability has been demonstrated by previous authors [25]. In this study, an increase in the temperature results in a significant decrease in the TMA yield. The heat effect was much more pronounced than that for the phenolic group. The maximum TMA yield was obtained at 28 °C and decreased by 50% at 30 °C and 88% at 32 °C.

### 2.4. Response Surface Analysis of Radical-Scavenging Activities

Table 2 presents the effect of independent variables on ABTS and DPPH radical-scavenging activities. The regression model was significant for both parameters at *p* < 0.001. ABTS activity was significantly affected by the quadratic effect of amplitude (*X*_1_^2^ = *p* < 0.01) and time (*X*_1_ = *p* < 0.01) and the linear and quadratic effect of time (*X*_2_, *X*_2_^2^; *p* < 0.01) (Figure 1c). The interaction between the amplitude with the time and the temperature (*X*_1_*X*_3_; *p* < 0.01; *X*_2_*X*_3_; *p* < 0.05) was also significant. The nonsignificant *p*-value of LOF test (*p* = 0.7814) demonstrated that the predicted model fits well to the data. Figure 2c presents the scatterplot of the predicted values versus the observed values. The adjusted R^2^ indicated that 90.7% of the variability was reported by the model. The final predictive equation obtained using significant terms for ABTS activity is expressed as follows:

*Y*_ABTS_ = −272.546 − 0.6540(*X*_2_) + 0.0188(*X*_1_^2^) − 0.0255 (*X*_2_^2^) − 0.0716 (*X*_1_*X*_3_) + 0.0707(*X*_2_*X*_3_) (3)

The effect of *X*_1_ (amplitude) and *X*_3_ (temperature) is displayed in 3D response surface contour plots (Equation (4); Figure 3c). Based on the desirability score of 1.0, the activity of ABTS was optimized at 38.79 μmol TE/g while calculating the model variables of 40% amplitude (28 W/cm^2^), 30 min, and 32 °C. An increase in ABTS was recorded with the increasing temperature at the optimum extraction time (Figure 4c).

The DPPH activity was significantly affected by the quadratic and linear effects of amplitude (*X*_1_^2^, *X*_1_; *p* < 0.01) and time (*X*_2_^2^, *X*_2_; *p* < 0.01). The interaction between the variables *X*_1_*X*_3_ and *X*_2_*X*_3_ was also significant (*p* < 0.01) (Figure 1d). The nonsignificant *p*-value of LOF test (*p* = 0.2538) demonstrated that the predicted model fits well to the data (Figure 2d). The adjusted R^2^ indicated that 86.52% of the variability can be shown by the model. The final predictive equation obtained using significant terms of the total antioxidant capacity is expressed as follows:

*Y*_DPPH_ = −539.26 + 1.323(*X*_1_) − 7.382(*X*_2_) + 0.0378(*X*_1_^2^) − 0.0785(*X*_2_^2^) − 0.1836 (*X*_1_*X*_3_) + 0.3875(*X*_2_*X*_3_) (4)

The effect of *X*_1_ (amplitude) and *X*_3_ (temperature) is displayed in 3D response surface contour plots (Equation (4); Figure 4d). Based on the desirability score of 1.0, the activity of DPPH was optimized at 62.27 μmol TE/g while calculating the model variables of 80% amplitude (74 W/cm^2^), 15 min, and 28 °C. DPPH recorded the same pattern as TPC and TMA, and increasing amplitude resulted in increased DPPH scavenging activity. Similarly, a further increase in time and temperature resulted in less DPPH activity.

DPPH was found to be a better indicator of the antioxidant activity of *B. alicastrum* than the ABTS, suggesting increased presence of anthocyanins, rather than another flavonoid. Under optimum conditions, the DPPH activity reached 65.74 μmol TE/g, which is a two-fold increase over the ABTS activity (38.79 μmol TE/g) measured at the point of the maximum desirability. The facts that flavonoids, tannins, and condensed tannins contribute to ABTS^+^ antioxidant capacity and anthocyanin contributes to DPPH have been reported earlier [26]. This study shows that the correlation between the observed values of ABTS and TMA (R = 0.868) was lower than that obtained between DPPH and TMA (R = 0.913), which reaffirms the results reported by the previous studies (Table 3). However, in contrast with Xu et al. [25] in this study, the correlation between ABTS and DPPH was also significant (R = 0.890), which is in agreement with that reported by Floegel et al. [27] who confirmed a high correlation that exists between the two radical-scavenging assays. As expected, the correlation between the observed TPC value and DPPH (R = 0.910) was also highly significant (Table 3). These correlation indices in some way validate the accuracy of the selected variables to optimize the extraction of *B. alicastrum* leaves.

## 3. Materials and Methods

### 3.1. Chemicals and Reagents

The chemical Gallic acid, 2,2-diphenyl-1-picrylhydrazyl radical (DPPH), 2,2-azinobs-3-ethylbenzothiazoline-6-sulphonic acid (ABTS), 6-hydroxy-2,5,7,8-tetramethylchromane-2-carboxylic acid (Trolox), and the Folin–Ciocalteu phenol reagent were purchased from Sigma-Aldrich (St. Louis, MO, USA). All chemicals used in the experiments were of analytical grade.

### 3.2. Plant Materials

Specifically, 1 kg of *B. alicastrum* leaves was collected during June and August from Yucatán State, México. The leaves were desiccated at 36 °C for 5 days in the laboratory (Convection Oven 107801, Fisher Scientific, Pittsburgh, PA, USA). To obtain a fine powder, dry leaves containing 16.9 ± 1.1% of moisture were ground in a manual mill, and then they were vacuum packed and stored at 4 °C.

### 3.3. Ultrasound-Assisted Extraction

The extractions were carried out in an ultrasonic processor by using a direct sonication method (VCX 130PB, Sonics, Milford, CT, USA). An ultrasound probe of 130 W (1/8′′ Probe) operating at 20 kHz was used for ultrasound treatment. Specifically, 2.0 g of dry *B. alicastrum* leaves was placed in a 50-mL glass tube containing 20 mL of 80% aqueous methanol. In order to maintain the stable temperature required for different treatments (28, 30, and 32 ± 0.5 °C), tubes were then positioned in a thermostatic recirculating water bath (VWR 117–612, St. Louis, MO, USA). The ultrasound probe was submerged to a depth of 30 mm in the samples; and by setting the amplitude of the probe, the energy input was adjusted. The amplitudes were set at 40%, 60%, and 80%, which correspond to a sonication power of 28 W/cm^2^, 51 W/cm^2^, 74 W/cm^2^, respectively. Each homogenate was sonicated for 10, 20, and 30 min, as the predetermined extraction time. Then, samples were centrifuged at 1200× *g* for 15 min at 10 °C (Eppendorf, 5804R, Westbury, NY, USA); and using the same procedure as described above, the sediment (7.25 ± 0.53 g) was subjected to an additional extraction. After finishing the second extraction, both supernatants were combined and the insoluble materials were removed by filtration by using a 0.45-μm membrane until a final volume of 25 mL was obtained. Finally, the extracts were kept in screw-capped dark tubes and stored at −20 °C for later use.

### 3.4. Total Phenolic Content (TPC)

The method, with some modifications, described by Singleton et al. [28] was used to measure TPC. Each extract with a quantity of 50 μL was mixed with 3 mL of ultrapure water (miliQH_2_O) and then 250 μL of 1N Folin–Ciocalteu reagent was added. After 5 min, 750 μL of 20% Na_2_CO_3_ and 950 μL of MilliQ H_2_O were added to the extracts. After 30 min of incubation at room temperature in a spectrophotometer at 765 nm, the absorbance was recorded (Vis-DR2700, Hach Company, Loveland, CO, USA). The concentration of TPC was calculated in units of milligram Gallic acid equivalents (GAE) per gram dry weight using a standard curve of Gallic acid (0–0.600 mg/mL) (Figure S1).

### 3.5. Total Monomeric Anthocyanin (TMA)

The pH differential method was used to measure TMA [29]. Briefly, to obtain the same dilution, each extract was diluted with buffers of pH 1.0 and pH 4.5. The absorbance was measured at 510 nm and 700 nm in both pH 1.0 and pH 4.5 buffers, respectively. Then, the following formula was used to calculate the TMA (expressed in terms of cyaniding-3-glucoside): TMA = (*A* × MW × DF × *V*_e_ × 1000)/ (*ε* × 1*M*), where *A* is the difference in the absorbance at (*A*_510_ − *A*_700_)_pH1.0_ − (*A*_510_ − *A*_700_)_pH4.5_, MW is the molecular weight of cyaniding-3-glucoside (449 g mol/g), DF is the dilution factor, *V*_e_ is the extraction volume, *ε* is the molar extinction coefficient of cyaniding–3-glucoside (29,600), and *M* is the mass of the extracted samples. The results were measured in units of milligrams of cyaniding-3-glucoside equivalent (CyE) per 100 g of dry weight.

### 3.6. Radical Scavenger Capacity (DPPH and ABTS Assays)

The antioxidant activities of the samples were analyzed by investigating their abilities to scavenging the DPPH and ABTS. The method of Re et al. [30] modified by Tachakittirungrod et al. [31] was used for the ABTS assay. The ABTS radical cation (ABTS^+^) was generated when the ABTS solution (7 mM) reacted with 2.45 mM potassium persulfate. The extracts (50 μL) or Trolox, as a reference substance, was allowed to react with 150 μL of the ABTS^+^ solution in the dark with known concentrations of Trolox. The decrease in absorbance at 750 nm was measured after 6 min by using a spectrophotometer (Multiskan EX, Thermo Scientific, Rockford, IL, USA). The ABTS scavenging activity was calculated in the units of micromoles of Trolox equivalents (TEs) per gram of sample (μmol TE/g) from the standard curve of Trolox (Figure S2).

The method of Brand-Williams et al. [32] was used to calculate the free radical-scavenging capacity of DPPH. Briefly, to obtain an absorbance of 0.560 ± 0.02 units, the solution of DPPH (0.1 mM) was diluted with 80% methanol. The extracts (0.1 mL) were reacted with 3.9 mL of the DPPH radical solution for 30 min in the dark, and the decrease in the absorbance of the reaction was measured at 541 nm (Multiskan EX, Thermo Scientific, Rockford, IL, USA). The radical-scavenging activity of extracts was calculated in units of micromoles of Trolox equivalents (TEs) per gram of sample (μmol TE/g) from the standard curve of Trolox.

### 3.7. Experimental Design for Optimization and Statistical Analysis

The process variables, such as sonication power (*X*_1_), extraction time (*X*_2_), and temperature (*X*_3_), involved in UAE of *B. alicastrum* leaves were optimized by RSM [33]. The experimental design, data analysis, and model building were analyzed by using the Statistics software (StatSoft, Inc., Tulsa, OK, USA). 

The BBD design assumed that the variables interact with each other and are measured using a second-order polynomial model as follows:

Y = β_o_ + Σβ_i_X_i_ + Σβ_ii_Xi^2^ + Σβ_ij_X_i_X_j_ (5)

In the second-order polynomial model, *Y* is the response value, βo is the constant, βi  is the linear regression coefficient, βii is the quadratic regression coefficient, βij is the interaction regression coefficient, and Xi and Xj are the independent variables. By evaluating the lack of fit (LOF) and the coefficient of determination (R^2^), the quality of fit of the polynomial model equation was determined. Using the F-test obtained from the analysis of variance (ANOVA), the significance of each coefficient was determined, and the effect was considered significant when it was above the standard error at the 95% confidence level (*p* < 0.05). The Pareto diagram was used to visualize the main effect of the factors and their interactions. By considering one response variable at its optimal level and plotting against two significant factors, three-dimensional response surface plots were generated. For the optimization process of each dependent variable (ip), the profile of the predicted values and the desirability analysis were used. The predicted values were optimized in a scale ranging from 0.0 (undesirable) to 1.0 (highly desirable).

## 4. Conclusions

Phenolic compounds have proven to have significant antioxidant activity, which is directly correlated with the removal of free radicals in the DPPH assay. Three variables, namely, time, temperature, and power were optimized by the BBD design, and the interaction between them was found to be highly significant for the optimization process. ANOVA statistics indicated that the sonication power plays a significant role in the yield of TPC and TMA. However, an increase in time and temperature significantly decreased the yield of phenolic compounds, particularly that of the TMA group. DPPH was found to be a better indicator of radical-scavenging activity of *B. alicastrum* leaf extract than ABTS. The optimization by the RSM technique confirmed that TPC and TMA groups of *B. alicastrum* enhanced the antioxidant activity of the phenolic compound, achieving better DPPH scavenging activity when submitted under conditions of low temperature (28 °C) and extraction time (less than 20 min) and higher sonication power (74 W/cm^2^).

## Figures and Tables

**Table 1:** Box–Behnken design (RSM 3-level-3-factor-1 replicate) and observed experimental total phenol content (TPC), total monomeric anthocyanin (TMA), and radical scavenging activity (ABTS and DPPH) data from *Brosimum alicastrum* left-extract.

Run	Block	*X_1_*		*X_2_*		*X_3_*		*Y_1_*	*Y_2_*	*Y_3_*	*Y_4_*
		Amplitude (%)		Time (min)		T° (±0.5 ^o^C)		TPC (mg GAE/g)	TMA (mg CyE)/100 g	ABTS (μmol TE/g)	DPPH (μmol TE/g)
1	1	−1	40	−1	10	0	30	35.67	3.76	27.57	31.04
2	1	1	80	−1	30	0	30	41.71	6.16	30.48	41.22
3	1	−1	40	1	30	0	30	41.82	7.41	31.47	42.66
4	1	1	80	1	30	0	30	42.86	6.99	35.06	45.54
5	1	−1	40	0	20	−1	28	39.03	5.22	29.74	38.01
6	1	1	80	0	20	−1	28	44.52	13.99	36.51	65.74
7	1	−1	40	0	20	1	32	42.65	8.14	33.59	43.60
8	1	1	80	0	20	1	32	40.82	5.53	29.95	39.12
9	1	0	60	−1	10	−1	28	34.85	3.44	22.96	25.44
10	1	0	60	1	30	−1	28	34.30	2.09	21.56	17.24
11	1	0	60	−1	10	1	32	33.97	1.88	19.50	14.23
12	1	0	60	1	30	1	32	37.82	4.70	26.54	36.99
13	1	0	60	0	20	0	30	36.68	3.24	25.86	31.66
14	1	0	60	0	20	0	30	38.79	4.80	27.86	38.60
15	1	0	60	0	20	0	30	36.71	3.97	26.24	32.95
1	2	−1	40	−1	10	0	30	36.13	4.35	27.88	35.92
2	2	1	80	−1	30	0	30	41.73	10.14	32.28	42.71
3	2	−1	40	1	30	0	30	42.31	10.51	37.56	50.03
4	2	1	80	1	30	0	30	44.36	10.09	35.34	51.42
5	2	−1	40	0	20	−1	28	39.58	5.51	31.81	45.28
6	2	1	80	0	20	−1	28	45.18	15.17	36.93	67.27
7	2	−1	40	0	20	1	32	43.06	10.50	38.79	44.13
8	2	1	80	0	20	1	32	41.75	6.27	31.42	39.58
9	2	0	60	−1	10	−1	28	35.39	5.07	23.23	28.48
10	2	0	60	1	30	−1	28	34.33	2.09	26.29	20.29
11	2	0	60	−1	10	1	32	34.11	2.47	21.04	17.28
12	2	0	60	1	30	1	32	37.91	6.02	26.97	39.01
13	2	0	60	0	20	0	30	36.93	3.68	25.98	36.50
14	2	0	60	0	20	0	30	39.47	5.10	30.53	38.88
15	2	0	60	0	20	0	30	38.48	4.56	26.88	33.16

TPC = Total phenolic content; TMA = Total monomeric anthocyanin; ABTS = 2,2′-azino-bis-3-ethylbenzthiazoline-6-sulphonic acid; DPPH = 2,2-diphenyl-1-picrylhydrazyl. Amplitude of 40%, 60% and 80% correspond to sonication power of 28 W/cm^2^; 51 W/cm^2^, 74 W/cm^2^, respectively.

**Table 2:** The analysis of variance for the response surface model of the independent variables of UAE for the *Brosimum alicastrum* left-extract.

	TPC (mg GAE/g)		TMA (mg CyE/100 g)		ABTS(μmol TE/g)		DPPH (μmol TE/g)	
	MS	*p*-Value	MS	*p*-Value	MS	*p*-Value	MS	*p*-Value
**Model**	36.3320	0.0000	32.0361	0.0000	75.6777	0.0000	429.957	0.0000
***Linear***								
***X_1_*** **Amplitude**	32.1503	0.0104 *	22.416	0.0008 **	5.7264	0.1397	239.85	0.0026 **
***X_2_*** **Time**	30.6911	0.0011 **	9.9792	0.0162 *	80.356	0.0000 **	279.59	0.0014 **
***X_3_*** **Temperature**	1.5012	0.3109	3.1125	0.1570	0.0956	0.8443	71.497	0.0746
***Quadratic***								
***X_1_*** **Amplitude**	174.427	0.0000 **	134.188	0.0000 **	418.97	0.0000 **	1688.89	0.0000 **
***X_2_*** **Time**	26.0715	0.0149 *	8.2767	0.0266 *	48.044	0.0003 **	454.96	0.0001 **
***X_3_*** **Temperature**	2.9281	0.2416	0.6913	0.4958	9.9678	0.0562	48.956	0.1351
***Interaction***								
***X_1_X_2_***	9.1465	0.0722 *	10.1941	0.0153 *	4.4310	0.1910	20.140	0.3293
***X_1_X_3_***	25.3343	0.0156 *	79.767	0.0000 **	65.538	0.0000 **	431.44	0.0002 **
***X_2_X_3_***	10.7341	0.0582 *	14.341	0.0051 **	16.005	0.0185 *	463.33	0.0001 **
**Lack of Fit (LOF)**	0.7945	0.8463	1.6661	0.1518	2.1285	0.7814	22.536	0.2538
**Pure error**	1.5523		0.5629		3.4676		10.943	
**R^2^ (RSU)**	0.9478		0.9172		0.9392		0.9116	
**Adj R^2^ (RSU)**	0.9203		0.8737		0.9072		0.8652	

TPC = Total phenolic content; TMA = Total monomeric anthocyanin; ABTS = 2,2′-azino-bis-3-ethylbenzthiazoline-6-sulphonic acid; DPPH = 2,2-diphenyl-1-picrylhydrazyl; * significance *p* < 0.05, ** significance *p* < 0.01.

**Table 3:** The significance of correlation coefficient from RSU of the total phenolic content (TPC), total monomeric anthocyanin (TMA) and radical scavenging activity (ABTS and DPPH) for the *Brosimum alicastrum* left-extract.

	Mean	SD	TPC	TMA	ABTS
**TPC**	39.09	3.46	1.000		
**TMA**	6.09	3.37	0.891 *	1.000	
**ABTS**	29.26	5.10	0.933 *	0.868 *	1.000
**DPPH**	37.80	12.20	0.910 *	0.913 *	0.890 *

* significance at *p* < 0.05.

## Supplementary Materials

The following are available online. Figure S1: Standard curve of Gallic acid, Figure S2: Standard curve of Trolox.
