# Supplementary figures and images for: Optimization of the Ultrasound-Assisted Extraction of Phenolic Compounds from Brosimum alicastrum Leaves and the Evaluation of Their Radical-Scavenging Activity
Source: Molecules. 2017 Aug 7;22(8):1286. doi: 10.3390/molecules22081286 (PMC6151988; doi:10.3390/molecules22081286)

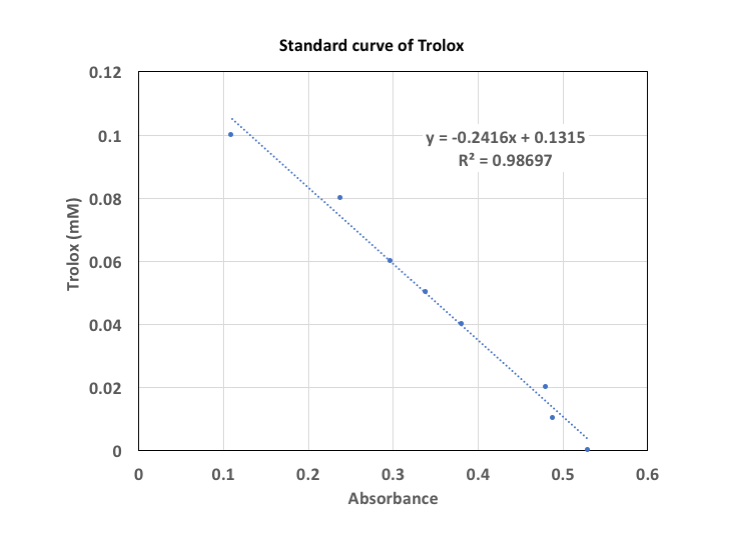

Supplement: Supplementary file 1 [file molecules-22-01286-s001.zip › S2. Standard curve of Trolox.tif]

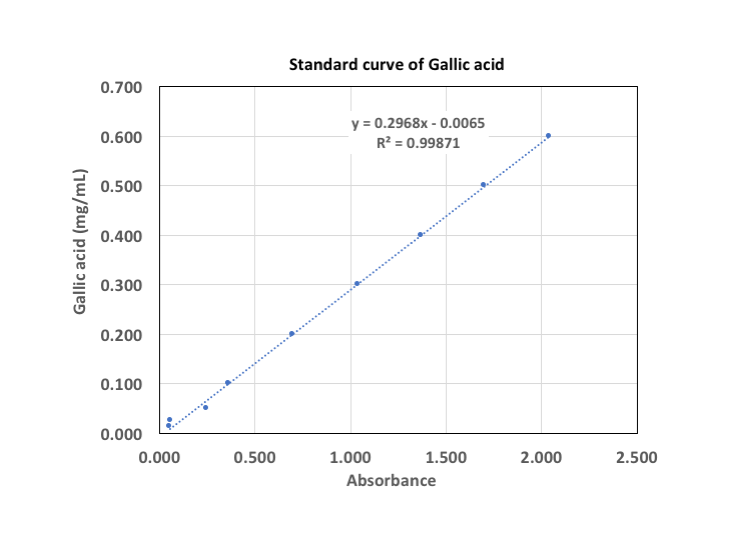

Supplement: Supplementary file 1 [file molecules-22-01286-s001.zip › S1.Standard curve of Gallic acid.tif]
